# Zika virus seroprevalence in women who gave birth during Zika virus outbreak in Brazil - a prospective observational study

**DOI:** 10.1016/j.heliyon.2020.e04817

**Published:** 2020-09-08

**Authors:** Lucas Victor Alves, Carla Adriana Leal, João Guilherme Bezerra Alves

**Affiliations:** a Department of Neuropediatrics, Instituto de Medicina Integral Prof. Fernando Figueira (IMIP), USA; b Department of Pediatrics, Instituto de Medicina Integral Prof. Fernando Figueira (IMIP), Brazil

**Keywords:** Epidemiology, Virology, Nervous System, Immune System, Laboratory Medicine, Zika virus, seroprevalence, pregnancy

## Abstract

**Background:**

The recent Zika virus (ZIKV) outbreak in Brazil started in August 2015 and ended in May 2017 without effective public health measures for its control have been taken. The immunological status of a community may not only predict future outbreaks as well to answer questions regarding ZIKV not known yet.

**Objective:**

To verify the seroprevalence of ZIKV in a group of women who were pregnant during the Zika virus outbreak in Recife, three to nine months after the delivery, and to evaluate the neurodevelopment of their children.

**Methods:**

A cross-sectional study enrolled participants of a cohort study held at Instituto de Medicina Integral Professor Fernando Figueira (IMIP) during the ZIKV outbreak in Recife. Mothers who gave birth between the last trimester of 2015 and the first semester of 2016, period of the peak of microcephaly outbreak in Recife, were invited. All participants had the serum tested by the anti-ZIKV IgG/IgM enzyme-liked immunosorbent assays, ELISA kit (Euroimmun, Lübeck, Germany). All children whose mothers presented positive serology for ZIKV performed the IgG/IgM ELISA test for ZIKV. These children were also evaluated by a neuropediatrician and the Denver II development screening test was applied.

**Results:**

Among the 132 studied pregnant women who gave birth at the peak of ZIKV outbreak in Recife, all were ZIKV IgM negative and 81 (61,3%) had ZIKV IgG positive. Mothers ZIKV IgG positive had more fever and rash during the pregnancy as compared with mothers negative for ZIKV; respectively 27/81 (33,3%) vs 6/51 (11,7%), p = 0.005 and 22/81 (27,2%) vs 4 (7,8%), p = 0.016. Only one child had IgG positive serology for ZIKV. No children had neurodevelopment defect for the age group and the Denver II normal scores.

**Conclusions:**

A high ZIKV IgG seroprevalence in pregnant women at the end of the ZIKV outbreak in Recife was found. This finding suggests that community protective immunity may have contributed to the end of ZIKV outbreak in Recife, Brazil.

## 1 Introduction

The recent Zika virus (ZIKV) outbreak in Brazil started in August 2015 in Recife, Pernambuco state [1]. At the time it was observed an unusual increase in newborns with microcephaly which was associated with Zika virus congenital infection [2]. The Ministry of Health declared microcephaly associated with ZIKV a national public health emergency in November 2015 and in February 2016 the World Health Organization declared ZIKV infection a public health emergency of international concern [3].

The ZIKV outbreak ended in Brazil in 2017 without effective public health measures for its control have been taken [4]. In Pernambuco, there were 433 cases of microcephaly associated with ZIKV during the Zika outbreak (2015–2016). Between 2017 and 2018, 12 cases of microcephaly and only one in 2019 [5]. At same time, other mosquito-borne, *Aedes aegypti*, infections such as dengue fever and Chikungunya remained high [6]. Most of the city of Recife continues without basic sanitation which makes the mosquito control almost impossible.

In Salvador, northeastern Brazil, it was demonstrated a high ZIKV infection rates which suggested that the ZIKV outbreak ceased due to community protective immunity [7]. It is important to know the immunological status for ZIKV not only to predict future outbreaks as well try to answer many knowledge gaps and research questions still need to be addressed. This study verified the seroprevalence of ZIKV in a group of women who were pregnant during the peak of ZIKV outbreak in Recife, three to nine months after delivery, and evaluated the neurodevelopment of their children.

## 2 Methods

### 2.1 Study population

We conducted a cohort study with pregnant women at Instituto de Medicina Integral Prof. Fernando Figueira (IMIP) during Zika virus outbreak in Recife, 2015–2016 [8]. This cohort study aimed to compared magnesium citrate with placebo in the prevention of adverse perinatal and maternal outcomes among pregnant women at higher risk.

**Figure 1:**
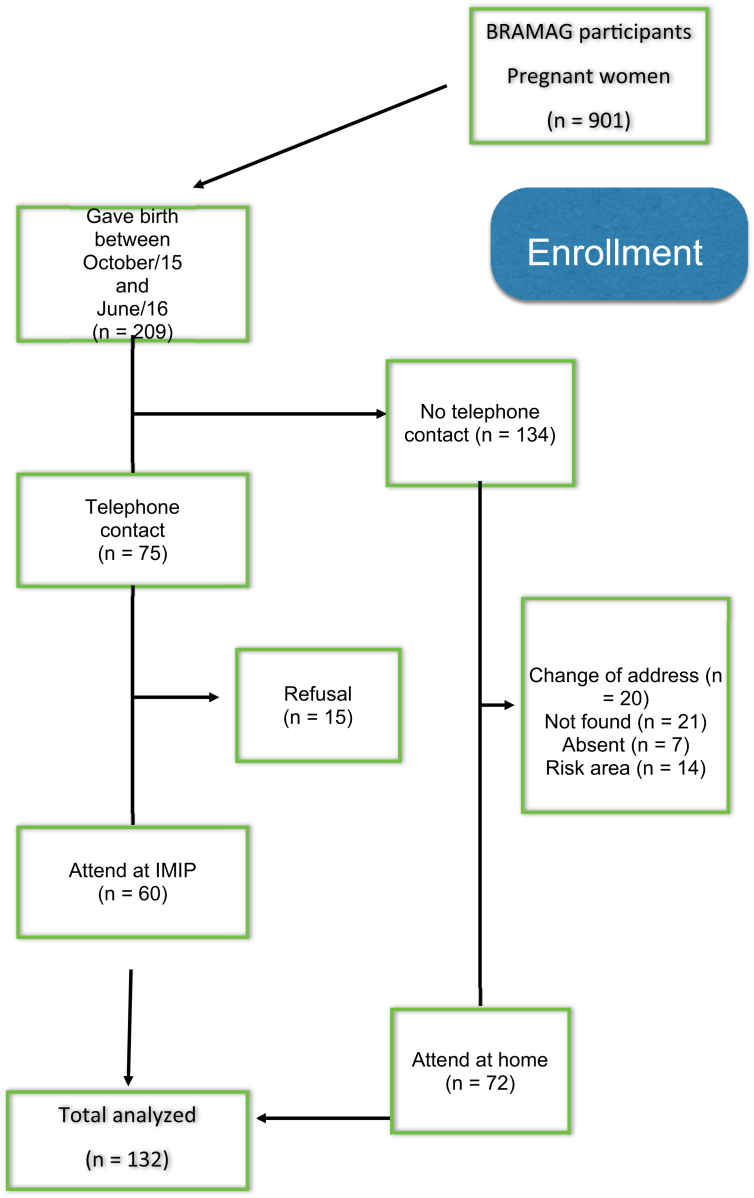
Flow diagram of the participants.

At that time 911 pregnant women had been enrolled and 209 participants gave birth between October 2015 and June 2016, peak of microcephaly outbreak in Recife. All these women were invited to participate in this study between three and nine months after their delivery. 132 women and their children met these criteria and were studied (Figure 1).

### 2.2 Sample and data collection

Recruited women who did not attend the call were visited at their homes. Participants were asked to fill out a short questionnaire asking about the neurodevelopment of their children. All the children of mothers who had a positive serology for ZIKV performed the IgG/IgM ELISA test for ZIKV. These children were also evaluated by a neuropediatrician and the Denver II development screening test was applied.

A total of 3 ml of blood drawn via venipuncture from each participant into an EDTA tube were transported to IMIP laboratory. Plasma samples were collected after centrifugation and stored frozen.

### 2.3 Laboratory assays

All the participants were tested by the anti-ZIKV IgG/IgM enzyme-linked immunosorbent assays, ELISA kit (Euroimmun, Lübeck, Germany), which uses the recombinant ZIKV-non-structural protein 1 (NS1) to minimize cross-reactivity to other flaviviruses. The measurement against a manufacturer-provided calibrator led to a ratio of extinction value for the sample to extinction value for the calibration solution. The manufacturer suggests interpreting the resulting ratios by classification into three categories based on ratio values (extinction_sample_/extinction_calibrator_): extinction ratio <0.8: negative; extinction ratio ≥0,8 ≤ 1,1: borderline; extinction rate >1,1: positive.

### 2.4 Study ethics

All the procedures followed were in accordance with the ethical standards of the Helsinki Declaration (1964, amended most recently in 2008). This project was previously approved by IMIP Ethical Committee of Research and all participants provided informed consent for their own and their child's participation.

### 2.5 Statistical analysis

All statistical analyses were performed with STATA/IC 12.1. Statistical association between ZIKV seropositivity and some participants characteristics were analyzed by t student and chi-square tests. A p value <0.005 was considered.

## 3 Results

Among the 132 studied pregnant women who gave birth at the peak of ZIKV outbreak associated with microcephaly in Recife, 2015–2016, 81 (61,3%), (95% CI: 52,9–69,7) had a positive serology IgG for ZIKV. Anti-ZIKV IgM was negative in all women studied. Some characteristics of the mothers according the ZIKV serology results are shown at Table 1. Mothers with positive ZIKV serology had more fever and rash during the pregnancy; respectively 27/81 (33,3%) vs 6/51 (15%), p = 0.05 and 22/81 (27,2%) vs 4/51 (7.5%), p = 0.016.

**Table 1:** Some characteristics of the 132 studied mothers according to the ZIKV serology.

	ZIKV positive (81)	ZIKV negative (51)	p
Fever during pregnancy	27 (33,3%)	6 (15%)	0.005
Fever period			
First trimester	11 (40,7%)	2 (33,3%)	0.736
After first trimester	16 (59,3%)	4 (66,7%)	
Fever after pregnancy	32 (39,5%)	17 (33,3%)	0.474
Rash during pregnancy	22 (27,2%)	4 (7,5%)	0.016
Rash period			
First trimester	10 (12,3%)	2 (50%)	0.101
After first trimester	12 (22,7%)	2 (50%)	
Rash after pregnancy	14 (17,2%)	8 (15,6%)	0.810
Use of repellents			
During pregnancy	47 (58%)	26 (50,9%)	0.427
After pregnancy	14 (17,2%)	9 (17,6%)	
Maternal age	27 (5,2%)	26,9 (4,8%)	0.919
Primipara	35 (43,3%)	21 (41,2%)	0.817
Marital status			
Married	49 (60,4%)	30 (58,8%)	0.848
Single/separate	32 (39,6%)	21 (41,2%)	

The children of the mothers with positive or negative ZIKV serology showed same APGAR score, birth weight and height and cerebral perimeter growth from birth to the period of study (Table 2). Serologic tests were collected between three and nine months after the birth (6,3 ± 32,2 months). Only one child from mothers with positive serology for ZIKV had IgG positive for ZIKV.

**Table 2:** Characteristics of the studied newborns according to ZIKV serology of their mothers.

	ZIKV positive (81)	ZIKV negative (51)	p
Gestational age (weeks)	38.6 (30–42)	38.6 (31–34)	0.828
Weight (g)	3,255 (1,9–4,4)	3,212 (1,135–4,164)	0.756
Height (cm)	48,4 (42–53)	48,2 (35–52)	0.746
Cephalic perimeter (cm)			
At birth	34,8 (42–53)	34,7 (33–37)	0.571
1 month	37 (35–39)	36,3 (35–39)	0.125
2 months	39,4 (36–41,5)	39,2 (37–42)	0.795
3 months	40,8 (39–44)	40,9 (39–43)	0.365
6 months	43,4 (41,5–46)	44 (41–46,5)	0.125
Apgar (5′) > 7	80 (98,7%)	51 (100%)	0.480

All these children presented a normal neurodevelopment for the age group. The results of the Denver II test were normal in all children.

## 4 Discussion

Our finding showed a high ZIKV infection rate in Recife at that time (61.3%), this high seroprevalence may suggests that the ZIKV outbreak ceased due to community protective immunity. Despite this outbreak has made effective public health responses challenging it seems unlikely that all the efforts made had played a key role on this outbreak control. The national response to the ZIKV outbreak has largely focused on household-level mosquito elimination efforts. These efforts may be futile without addressing systemic problems with public infrastructure, such as limited access to piped water and poor sanitation [9]. Such conditions provide ideal mosquito habitats, which exacerbated the recent ZIKV outbreak, as well as outbreaks of other arboviruses in Brazil. This puts both inhabitants and travelers at risk [10] because ZIKV infection during pregnancy is a known cause of microcephaly and other congenital and developmental anomalies [11]. It seems a very difficult task to control the mosquito (*Aedes aegipty*) in a region without basic sanitation such as Recife. In addition, other mosquito-borne arboviruses as Chikungunya continued to have a high incidence in Brazil [6].

The high ZIKV seroprevalence observed in this study was very similar to that detected in Salvador, 63%, also in northeast of Brazil [7]. ZIKV outbreak had very similar characteristics in Recife and Salvador. During the ZIKV epidemic in French Polynesia (the outbreak began in October 2013, peaked in December 2013 and was over in April 2014) the ZIKV seroprevalence increased to 50% (95% CI: 43% —incr%; 97/196) by the second half of the outbreak and reached 66% (95% CI: 62%–70%; 314/476) after the end of the outbreak [12]. All these findings suggest a rapid spread and development of immunity of ZIKV outbreak which competes for a relative short time duration of ZIKV outbreak.

Fever and rash during pregnancy were more frequent in studied women with ZIKV positive serology. But none of the studied children showed alterations in their neurodevelopment around two years of age and all of them had a normal Denver II test scores. This seems to indicate that these children were free of neurologic damage caused by ZIKV in case if they had ZIKV congenital infection. There is a concern of infants born without obvious structural brain abnormalities following in utero ZIKV exposure would have a normal neurodevelopment. Two studies showed that about 14% of children born with ZIKV congenital infection had some neurodevelopment alterations [13]. Recently, it was showed alterations in body composition in the ZIKV exposed in pregnancy infants at 3 month of age [14].

Only one studied child showed IgG positive ZIKV serology. This child also has a normal neurologic development including visual and audiological assessments. Trans placental transfer of ZIKV-specific IgG in pregnancy could contribute to protection of the fetus from congenital Zika syndrome. IgG levels may persist over a long time and may not be reflective of recent infection. The high ZIKV seroprevalence in mothers and very low in their children can be interpreted initially as the mothers became infected by ZIKV after childbirth. However, it was a very short time after childbirth, three to nine months, to the mothers get ZIKV infection. The fetal immune response to ZIKV is not yet completely well known. Recently it was showed that seroconversion of ZIKV-IgG may be observed at 21 months of age. The passage of antibodies through breast milk is also unknown. A seroprevalence study of ZIKV in Saudi Arabia among asymptomatic pregnant mothers and their newborns found IgG ZIKV in 12,6% of the mothers but all infant samples were negative [15]. The fetal immune response to ZIKV infection needs to be further studied.

The strengths of our study include a large sample from pregnant women during the ZIKV outbreak and a complete and longitudinal analysis of mother-child pair after the Zika outbreak. As limitations, the serological test were performed around six months after childbirth which generates the hypothesis of ZIKV infection post-birth. Besides some doubts remain about cross-reactivity among flaviviruses [16]. However, the ZIKV non-structural (NS1) protein has been identified as being largely specific to the virus and the ELISA method is considered as an adequate approach to detect ZIKV-specific IgM and IgG [17]. In a previous study, 24 children with congenital Zika syndrome born with microcephaly during the Zika outbreak in Brazil in 2015, all had a serum sample positive for IgM anti-Zika [18]. Nevertheless, among several commercial kits available for direct viral detection by nucleic acid-based testing the Euroimmun Zika IgG has a sensitivity of 71% and specificity 70% [19] and has been used in ZIKV serological studies in various regions of the world.

In conclusion, we detected a high ZIKV IgG seroprevalence in pregnant women studied during the ZIKV outbreak in Recife, around six months after the delivery. Although the sample size studied is not representative of Recife city, this finding may suggest that community protective immunity could restrict ZIKV spread and contributed to the end of ZIKV outbreak. IgG seropositivity has been associated with lasting long-term protection against ZIKV infection. Despite this high IgG ZIKV seroprevalence and more clinical symptoms compatible with ZIKV infection (fever and rash) have been observed in positive IgG ZIKV women, their children had ZIKV negative serology, except one child, and all had normal neurodevelopment for age. Further studies are needed to verify the fetal immune response to ZIKV and clarify the effect of ZIKV intrauterine infection on neurodevelopment of children born without the congenital Zika syndrome.

## Declarations

### Author contribution statement

L.V. Alves, C.A. Leal, J.G. Alves: Conceived and designed the experiments; L.V. Alves, C.A. Leal: Performed the experiments; L.V. Alves, J.G. Alves: Analyzed and interpreted the data; L.V. Alves: Contributed reagents, materials, analysis tools or data; L.V. Alves, J.G. Alves: Wrote the paper.

### Funding statement

This work was supported by supported by 10.13039/501100003593CNPq and 10.13039/100000865Bill & Melinda Gates Foundation (Grant number 4399862016-8, OPP1107597).

### Competing interest statement

The authors declare no conflict of interest.

### Additional information

No additional information is available for this paper.

## Ethical approval

All the procedures followed were in accordance with the ethical standards of the Helsinki Declaration (1964, amended most recently in 2008). This project was previously approved by IMIP Ethical Committee of Research and all participants provided informed consent for their own and this child's participation.
